# Th2 cells and cytokine networks in allergic inflammation of the lung

**DOI:** 10.1155/S096293519500038X

**Published:** 1995-07

**Authors:** Anthony J. Coyle, Shogo Tsuyuki

**Affiliations:** 1 Asthma and Allergy Research Department CIBA-GEIGY AG Basel CH-4002 Switzerland; 2 Dept. of Research Kissei Pharmaceutical Nagano Prefecture Matsumoto Japan

## Abstract

The cytokines released from Th2 and Th2-like cells are likely to be central to the pathophysiolgy of asthma and allergy, contributing to aberrant IgE production, eosinophilia and, perhaps, mucosal susceptibility to viral infection. IL-4 has emerged as a central target, not only for B cell IgE production, but also in the commitment of both CD4+ and CD8+ T cells to cells with Th2 effector function capable of secreting IL-5 resultlng in eosinophilic inflammation. In view of the central role of this cytokine and the evidence that glucocorticoids are unable to modify many IL-4 dependent effects, Th2 inhibitors may prove to be novel therapies for the treatment of bronchial asthma.


Mediators of Inflammation 4, 239–247 (1995)

### Th2 cells and cytokine networks in allergic inflammation of the lung

**Anthony J. Coyle[1,CA] and Shogo Tsuyuki[2]**

[1]Asthma and Allergy Research Department, CIBA-GEIGY AG, Basel, CH-4002, Switzerland; [2]Dept. of Research, Kissei Pharmaceutical, Matsumoto, Nagano Prefecture, Japan

[CA]Corresponding Author

**The cytokines released from Th2 and Th2-like cells are likely to be central to the pathophysiology of asthma and allergy, contributing to aberrant IgE production, eosinophilia and, perhaps, mucosal susceptibility to viral infection. IL-4 has emerged as a central target, not only for B cell IgE production, but also in the commitment of both CD4+ and CD8+ T cells to cells with Th2 effector function capable of secreting IL-5 resulting in eosinophilic inflammation. In view of the central role of this cytokine and the evidence that glucocorticoids are unable to modify many IL-4 dependent effects, Th2 inhibitors may prove to be novel therapies for the treatment of bronchial asthma.**

**Key words:** Cytokines, Inflammation, Lung, Th2 cells.

### Introduction

There is now increasing evidence to suggest that pro-inflammatory cytokines derived from various subsets of T cells play a major role in the induction of allergic inflammation of the lungs. Additionally, cytokines derived from other cell types such as mast cells and eosinophils may also be of importance in the maintenance/persistence of this disease. This review discusses the interactive cytokine networks that exist in the lungs, which are believed to play an important role in the aetiology of bronchial asthma.

### Pathology of asthma

Bronchial hyperresponsiveness (BHR) to both specific and nonspecific stimuli is a characteristic feature of bronchial asthma. While the mechanisms underlying this exaggerated responsiveness are still unclear, there is a considerable body of evidence to suggest that mucosal inflammation of the airways is of central importance. Perhaps the most common pathological finding is an increased number of eosinophils and mast cells in the lung mucosa.[1] It is currently believed that damage to the epithelium is mediated by the secretion of eosinophil-derived highly toxic cationic proteins such as major basic protein (MBP). In addition, ultrastructural abnormalities in the architecture of the lungs of asthmatic individuals have been reported, characterized by subepithelial deposition of collagen types III and V and fibronectin, the amount of which is correlated with the number of myofibroblasts. More recently, attention has been focused on the role of lymphocytes in the pathophysiology of this disease. In severe acute asthma, an increase in the number of T cells expressing the activation markers HLA-DR, CD25 and VLA-1 has been reported.[2] The realization that CD4+ T cells can be committed to a distinct phenotype that can induce B cells to switch to IgE production and recruit eosinophils to the lungs, mediated via the secretion of cytokines, has led to the hypothesis that lymphocytes play a major role in orchestrating the inflammatory response in the lungs of asthmatic individuals.

### CD4+ T cell subsets—the Th2 hypothesis

Mature T cells in the periphery can be divided into either CD4+ and CD8+ populations. This subdivision is associated with fundamental differences in their function, in that CD4+ T cells are activated by soluble foreign proteins such as allergens, presented by MHC class II molecules on the cell surface of antigen presenting cells (B cells, macrophages and dendritic cells). In contrast, CD8+ T cells are, in general, activated by intracellular pathogens such as viruses, are MHC class I restricted and are cytotoxic. Murine CD4+ T cells can be further subdivided into two distinct subsets, termed T helper 1 (Th1) and T helper 2 (Th2), on the basis of their restricted cytokine profile and ability to mediate different immune functions.[3] Activated antigen-naive resting CD4+ T cell cells secrete mainly IL-2. However, these cells can be primed under the influence of different cytokines (and perhaps dif-

© 1995 Rapid Science Publishers ferent co-stimulatory signals) to either a Th1 or Th2 subset. Th1 cells produce TNF-β and IFN-γ and are involved in delayed hypersensitivity responses. Th2 cells produce IL-4, IL-5 and IL-10 and provide help to B cells and as such, are believed to play a central role in the aetiology of allergic disease.[3]

### Th2 cells in asthma

While the concept of Th2 subtype of CD4+ T cells was originally defined in murine cell clones, there is now evidence to suggest that a similar cytokine profile exists in individuals with allergic disease. Studies of T cell clones obtained from the blood of patients sensitive to house dust mite allergen demonstrated that the majority of these clones produced IL-4 and IL-5 and low levels of IFN-γ and IL-2 after antigen stimulation.[4] Likewise, lymphocytes obtained by bronchoalveolar lavage after allergen provocation of allergic individuals have increased expression of mRNA for IL-3, IL-4 and IL-5.[5] IL-5 mRNA has also been identified by *in situ* hybridization of tissues obtained by bronchial biopsies of asthmatic subjects and localized to beneath the epithelial basement membrane.[6,7] However, it is important to realize that the Th1/Th2 concept is based on extremely polarized panels of cytokines which can be generated *in vitro* by murine CD4+ T cells and it is likely that more subtle intermediate phenotypes exist in human disease.

### Antigen specific activation of CD4+ T cells

Activation of CD4+ T cells require interaction between CD3 associated TCR α/β complex and specific antigen presented as peptides by class II bearing cells. However, for complete cellular activation, a second signal is also required. Moreover, antigenic stimulation through the TCR in the absence of co-stimulation leads to a state of unresponsiveness or clonal anergy. The most extensively studied co-stimulatory molecule is CD28, which is constituatively expressed on the surface of naive CD4+ T cells, the engagement of which is required for IL-2 production and T cell proliferation.[8] The importance of this pathway is illustrated in mice lacking the gene for CD28 which exhibit impaired lymphokine secretion.[9] The first ligand for CD28, initially termed B7 (now classified as CD80), was identified on the surface of the B cell and subsequently shown to provide co-stimulatory signals to antigen activated T cells.[10,11] However, the demonstration that B7-knockout mice had virtually no immune defects led to the speculation that additional CD28 ligands existed.[12] This indeed proved to be the case and a second co-stimulatory molecule termed B7-2 (CD86) has more recently been identified.[13] B7-2 is expressed at extremely low levels on resting B cells, but is rapidly upregulated after cross linking of the B cell antigen receptor or following stimulation with LPS.[14] On human B cells, B7-2 expression peaks within 24 h of activation, whereas B7-1 expression peaks several days later.[15] In addition, a second CD4+ T cell co-receptor, homologous to CD28 has been identified, termed CTLA-4 and demonstrated to be a second ligand for B7-1 and B7-2.[16] The different functional importance of CD28 and CTLA-4 is still unclear. However, in contrast to CD28, CTLA-4 expression is increased only after activation,[11] and as such may function to downregulate the T cell response.[17]

While the importance of these co-stimulatory signals for the production of IL-2 is well established, the role of these co-stimulatory molecules in the production of Th2 pattern of cytokines is less clear. However, it has recently been reported that stimulation through CD28 induces a Th2 response *in vitro* independent of IL-4 production.[18] CD28 mediated stimulation of cloned human Th2 clones has also been reported to induce responsiveness to IL-4 via the autocrine production of IL-1α.[19] *In vivo*, administration of CTLA-4Ig can inhibit IL-4 gene expression and IgE production following infection with *Heligmosomoides polygyrus*.[20] Further evidence that co-stimulation modifies the outcome of an immune response is provided by the observation that treatment with CTLA-4 Ig prevents IL-4 production following immunization with goat-anti-IgD.[21] In contrast, mice transgenic for soluble murine CTLA-4IG exhibit normal T cell priming and cytokine production after immunization with a T cell dependent antigen.[22] The reasons for these discrepancies are unclear, but may be related to insufficient levels of CTLA-4Ig *in vivo* to block interactions between APC and T cells.

### Cytokines and allergic inflammation

*Regulation of IgE production:* The principal feature that distinguishes atopic from non-atopic individuals is their ability to develop IgE antibodies to foreign proteins. During a CD4-MHC class II dependent cognate interaction of T and B cells, contact-mediated costimulatory signals provided by CD4+ T cells such as CD40L and membrane TNF-α, initiate B cell activation. During this process, IL-4 has been demonstrated in murine[23,24] and human[25,26] systems to instruct B cells to switch to IgE production. Investigation of T cell cytokine production indicates that enhanced *in vitro* IL-4 production is correlated with enhanced *in vivo* IgE production. Whereas T cell membrane-dependent activation signals are regarded as general competence signals to initiate B cell activation and induce cytokine responsiveness, cytokines such as IL-4 provide a progressive signal inducing proliferation and switching of B cells to IgE. Inhibition of IL-4 *in vivo*, either by administration of anti-IL-4 antibodies[24] or by deletion of the IL-4 gene,[27] has revealed the essential requirement of IL-4 for the switch to IgE production. However, a recent study has demonstrated that IL-4 independent IgE production can occur, although at a greatly reduced level in IL-4 gene targeted mice.[28] However, to what degree such production might be mediated by already switched B cells remains to be determined. The situation, however, is more complex in humans as IL-13, the message of which has been found in murine and human CD4+ cell clones of Th0 and Th2 phenotype, as well as CD8 T cell clones,[29] can also induce immunoglobulin isotype switching to IgE production.[30] The precise importance of IL-4 *vs.* IL-13 as the principal cytokine involved in B cell isotope switch in allergic disease is at present unclear. However, recent studies have demonstrated that naive CD4+ T cells (CD45RO−) primed through the TCR develop into effector cells that secrete IL-5 and IFN-γ and help B cell IgE production via IL-13 and not IL-4.[31] In contrast, activation of CD4+ memory cells (CD45RO+) also produce IL-4 and IL-5 and help B cell IgE through a combination of IL-13 and IL-4.[31]

IL-4 also plays a central role in the induction of the Th2 phenotype as shown by *in vitro* and *in vivo* experiments using IL-4 gene detected mice. The importance of IL-4 in the cytokine networks in the lung are discussed in more detail in the next section.

*Development of eosinophilic inflammation:* Over 20 years ago, the development of eosinophilia in nematode infected rodents was demonstrated to be lymphocyte dependent.[32] Subsequently, it was shown that the soluble factor from T cells was identical to B cell growth factor 2 (BCF II). This factor has been extensively studied and characterized and is now termed interleukin-5 (IL-5). IL-5 has been shown to promote the growth and differentiation of eosinophils in culture and, in contrast to IL-3 and GM-CSF, acts as a terminal eosinophil differentiation factor. *In vivo*, administration of exogenous IL-5 induces eosinophil recruitment[33] and transgenic mice overexpressing the IL-5 gene develop peripheral blood, bone marrow and tissue eosinophilia.[34] Furthermore, administration of anti-IL-5 has been demonstrated to inhibit eosinophilia induced by nematodes[35] or antigen in sensitized animals.[36] The precise source of IL-5 required for the induction of eosinophil infiltration is at present unclear, although it is likely that it is produced from CD4+ T cells. IL-5 (in addition to IL-3 and GM–CSF) is also required for the survival of eosinophils *in vitro*.[37,38] However, in contrast to the requirement for CD4+ T cell derived IL-5 for the recruitment of eosinophils to the lung,[39] the source of IL-5 required for the survival of eosinophils in the lungs is from a non-CD4+ T cell source, possibly involving autocrine production from the eosinophil itself.[40] IL-5 however, may not be the only factor involved in recruiting eosinophils to the mucosa and/or promoting their survival and activation, as the observation of a complete inhibition of eosinophil infiltration into the airways after antigen challenge in mice lacking the IL-5 gene (M. Kopf, personal communication) may also reflect the requirement for maturation of eosinophils in the bone marrow. In this regard, other cytokines including the chemokines RANTES and the recently identified eotaxin, may also be important in recruiting eosinophils to the lung.

RANTES, along with IL-8, MCP-1, MCAF and eotaxin belong to the superfamily of structurally related low molecular weight cytokines which contain four cysteines at identical relative positions with a conserved Cys–Cys– (C–C) motif. RANTES was originally identified as an apparently T cell-specific inducible gene that was expressed by cultured T cell lines.[41] More recently, RANTES has been shown to be produced from activated platelets.[42] RANTES is eosinophil chemotactic for both eosinophils and for 'memory' CD4+ T cells *in vitro* and as such may contribute to the eosinophil recruitment in allergic disease.[43]

Eotaxin, was originally identified as a factor produced in the BAL of antigen-challenged guinea-pigs, which, upon subsequent injection, induced a selective accumulation of eosinophils in the skin[44] and lungs.[45] Eotaxin has a 53% homology with MCP-1, 31% with MIP 1α and 26% with RANTES. mRNA for eotaxin was shown to be up-regulated 3 h after allergen provocation.[46] The role of eotaxin and other C–C chemokines in the airways of asthmatic individuals remains to be determined.

*CD8+ Th2 like cells—a mechanism for viral used exacerbations of asthma:* CD8+ T cells typically produce a Th1 like cytokine panel (IFN-

γ, IL-2) after *in vitro* stimulation. CD8+ T cells mediate lysis of viral infected cells and inhibition of viral replication through the production of IFN-γ. Moreover, it has been suggested that CD8+ T cells down-regulate the CD4+ T cell driven response dependent on the production of IFN-γ.[47,48] However, recent observations from Erard and colleagues have shown that in the presence of IL-4, activated CD8+ cells can switch their function to produce Th2 cytokines.[49] Additionally, these cells produce less IFN-γ and lose their cytotoxic potential. This suggests that in allergic individuals, CD8+ T cell function may be re-directed if activated by a specific antigen in the presence of IL-4. In this context, it has recently been demonstrated that MHC class I restricted viral antigen-specific activation of CD8+ T cells in the presence of IL-4, leads to a phenotype that produces IL-5 and reduced amounts of IFN-γ.[50] Moreover, *in vivo*, the induction of an IL-4-dependent Th2 phenotype can switch viral-specific CD8+ T cells to produce IL-5 and induce eosinophil recruitment into the lungs.[50] These observations suggest that IL-4 is important not only in regulation of CD4+ T cell commitment, but has dramatic effects on CD8+ T cell function. Thus, this IL-4 mediated switch of CD8+ T cells to a Th2 like phenotype, may not only exacerbate asthma severity by secreting IL-5, but the reduction in IFN-γ secretion may impair the normal host response, leading to delayed viral clearance from the lung.

*IL-4 and steroid resistant asthma:* Glucocorticosteroids are the single most effective class of drug able to control symptoms and suppress overt airway inflammation in asthma, and are increasingly widely used as first line therapy. It has recently been reported that treatment of allergic individuals with systemic prednisolone inhibits the expression of mRNA for IL-4 and IL-5 mRNA, whilst increasing mRNA for IFN-γ, suggesting that one of the therapeutic effects of this drug is to inhibit Th2 cell activation.[51] Steroids may also be effective by inducing apoptosis of eosinophils in the inflamed mucosa, by suppressing IL-3, GM-CSF and IL-5 synthesis.[52]

However, steroids are unable to suppress IL-4 induced up-regulation of the endothelial adhesion ligand for eosinophils, and VCAM-1[53] and can enhance IL-4-driven IgE production in normal human lymphocytes. Furthermore, in a Th2-like T cell line, IL-4 selectively inhibits dexamethasone-induced apoptosis. In this context, it has recently been demonstrated that *in vitro* steroids are unable to inhibit IL-4 secretion from murine Th2 cells.[54] Together, these observations suggest that steroids may not be able to inhibit some of the effects and the production of IL-4, and may therefore be intrinsically unable to suppress the central underlying immunological processes that drive eosinophilic inflammation in the mucosa.

More recently, it has been shown that prednisolone therapy fails to down-regulate mRNA for IL-4 and IL-5 in steroid resistant asthmatics.[55] While the precise mechanisms by which steroid resistance occurs is unclear, it has recently been demonstrated that IL-4 can down-regulate the glucocorticoid receptor affinity *in vitro*.[56] These observations suggest that inhibition of IL-4 may, in addition to providing an alternative therapy to steroids, prove to be a useful adjunct therapy in the treatment of asthma by possibly restoring normal steroid receptor function.

### Cytokine regulation of Th1 and Th2 cell commitment

Activated antigen-naive resting CD4+ T cell cells from non-allergic donors secrete mainly IL-2, with a very low production of IL-4 and IFN-γ. These cells must be 'primed' or 'committed' to either a Th1 (IFN-γ producing) or Th2 (IL-4 producing) phenotype. It is now well established *in vitro* that IL-4 is essential for the commitment of naive CD4+ T cells to the Th2 phenotype after either stimulation through the CD3 complex[57,58] or by a specific antigen.[59] Likewise, studies performed in mice lacking the IL-4 gene have demonstrated the essential role of this cytokine in the development of Th2 immune response, as *ex vivo* stimulation of CD4+ T cells from these mice causes the secretion of greatly reduced amounts of IL-5 and IL-10, and fail to mount an IgE response. Likewise, these mice have a marked attenuation of IL-5 dependent lung eosinophil recruitment following aeroallergen provocation. The initial source of IL-4 required for Th2 commitment is at present uncertain. As discussed below, mast cells and basophils can produce IL-4 following cross-linking of IgE bound to the FcεR1.[60] However, it is unlikely that mast cells would function as the primary source of IL-4 as in murine systems IgE production is strictly IL-4 dependent. Moreover, adoptive transfer of CD4+ T cells alone from normal mice to IL-4 gene deleted mice results in the development of an IgE response following immunization, suggesting that for the induction of the B cell isotype switch to IgE, IL-4 is derived solely from CD4+ T cells.[61] More recently, a small subpopulation of T cells derived from the spleen and bearing the markers

CD4+/NK1.1+ have been shown to rapidly produce IL-4 mRNA upon activation, independent of IL-4 itself.[62] These observations suggest that possibly these cells can provide the first source of IL-4 at the outset of the immune response which subsequently primes naive CD4+ T cells to the Th2 phenotype.

IL-10 is a major cytokine produced by several different cell types. Mouse IL-10 is secreted from Th2, but not Th1 cells.[63] In addition, mouse CD5+ B cells produce IL-10 and it has been suggested that autocrine production of IL-10 by these cells acts as a growth factor.[64] Murine macrophages are also a source of IL-10. In contrast, the production of IL-10 by human cells is not restricted to Th2 cells, and approximately one third of CD8+ T cell clones, as well as Th0 and Th1 cells, produce IL-10 upon restimulation.[65] In comparison to other cytokines, IL-10 is synthesized at a late stage after activation, with maximal expression of mRNA 24 h and protein production 48 h later.[65] IL-10 inhibits production of IFN-γ and the proliferation of Th1, but not Th2 cells.[63] Moreover, inhibition was observed only in the presence of macrophages and not B cells, suggesting an indirect mode of action via the antigen-presenting cell itself.[66] This inhibition appears to be unrelated to antigen processing, as IL-10 also inhibited cytokine production from Th1 cells induced by superantigens.[65] IL-10 is a potent suppresser of macrophage activation and inhibits the production of IL-1α, IL-1β, IL-6, IL-8, TNF-α and GM–CSF from monocytes at both the protein and transcriptional level. IL-10, like IL-4, up-regulates class II MHC expression on small resting B cells and maintains their viability *in vitro*, although in contrast to IL-4, IL-10 failed to increase expression of CD23. Thus, IL-10 appears to down-regulate the immune response by inhibiting the production of pro-inflammatory cytokines. In this context, it has recently been demonstrated that IL-10 will inhibit the accumulation of eosinophils in the lung after antigen challenge, associated with a supression of TNF-α in the bronchoalveolar lavage fluid.[67]

IFN-γ has been shown to inhibit proliferation of Th2 cells and to inhibit IL-4 induced Th2 phenotype commitment.[68] Furthermore, IFN-γ has been shown to prime cells for further IFN-γ production. *In vivo*, IFN-γ has been shown to inhibit the recruitment of eosinophils to the lungs of sensitized mice.[69] Moreover, anti-IFN-γ potentiated the antigen-induced eosinophil accumulation, suggesting that endogenous IFN-γ secreted following aerosol antigen provocation acts to inhibit mucosal Th2 immune responses.[69] It has therefore been proposed that the balance between IL-4 and IFN-γ production will determine whether a Th1 or a Th2 phenotype is produced. This cross regulation has been elegantly demonstrated in models of parasite infection where neutralizing antibodies against IL-4 or IFN-γ have been used to resolve or promote infection by biasing the immune response in favour of Th1 or Th2 immunity.

IL-12 is also believed to play a central role in the regulation of the immune response. IL-12, like TNF-α, IL-1 and IL-6, is secreted principally from macrophages in response to bacterial or viral pathogens. IL-12 has been reported to facilitate the development of the Th1 phenotype, efficiently prime cells for IFN-γ production and inhibit the commitment of IL-4 producing cells.[70] Similarly *in vivo*, IL-12 shifts the immune response from an IL-4 producing to an IFN-γ producing phenotype and provides protection against *Leishmania major* infection where Th2 responses are lethal.[71] More recently, it has been demonstrated that IL-12 will inhibit IL-4, IL-5, IL-6 and IL-13 mRNA and potentiate expression of mRNA for IFN-γ, IL-2 and IL-10 after infection with *Schistosoma mansoni*.[72] Studies using neutralizing antibodies to IFN-γ demonstrated that the effects of IL-12 on expression of IL-2, IL-4 and IL-13 were mediated through IFN-γ production. However, the suppressive effects of IL-12 on mRNA for IL-5 and IL-6 were relatively refractory to IFN-γ[72] raising the possibility that IL-12 may be a more important regulator of IL-5 production than IFN-γ. In human T cells, IL-12 can switch the *in vitro* recall response of allergen-specific T cells from atopic donors, from a Th2 to a Th1 phenotype, suggesting that IL-12 can not only commit cells to a Th1 phenotype, but also has the potential to reverse established immune response.[73]

IFN-α has a wide range of immunomodulatory activity and has been shown to suppress IgE production both *in vivo*[74] and *in vitro*.[75] However, a direct effect on B cells is unlikely as IFN-α fails to inhibit B cell switch *in vitro* unless IFN-γ is present.[76] The hypothesis that IFN-α mediates its suppressive effect via IFN-γ production is supported by the finding that IFN-α induced inhibition of anti-IgD induced IgE and IgG1 production is associated with an increase in splenic mRNA for IFN-γ.[77] This hypothesis has recently been supported using human CD4+ T cells, showing IFN-α increases the frequency of IFN-γ producing CD4+ T cells and antagonizes the suppressive effects of IL-4 on IFN-γ production.[78] As a consequence, both IL-12 IFN-γ and IFN-α may favour the induction and maintenance of Th1 cells and counteract Th2 driven allergic reactions (Fig. 1).

### Cytokines from a non-CD4+ T cell source

*Mast cells and basophils:* Mast cell metaplasia in the bronchial mucosa is a pathological feature of chronic asthma. Mast cells have been shown to be a rich source of cytokines and as such may not only contribute to the acute responses following antigen challenge, but may also play an important role in the persistence of airway inflammation. Murine non-B/non-T cells (either mast cells or basophils) have been reported to secrete IL-4 and IL-6,[79] as well as TNF-α and GM–CSF upon cross linking of the Fcε receptor.[79] Likewise, human mast cells also secrete IL-4 upon IgE specific activation.[60] More recently, it has been shown that murine mast cells can also synthesize and secrete IL-13 upon IgE dependent activation and induce IgCε transcripts.[80] Additionally, mast cells produce IL-3, which together with IL-5, facilitate expansion of the eosinophil lineage and enhance eosinophil survival. IL-3 can also prime mast cells for cytokine production (Table 1). Metachromatic cells may therefore have an important role as rapid sources of cytokines triggered by IgE-dependent cell activation. Indeed, a positive feedback loop may operate involving IgE, mast cells and Th2 cytokines derived from CD4+ T cells. Thus, antigen exposure would lead to the production of IL-4 from CD4+ T cells which would then express CD40L upon activation and facilitate the production of IgE, which in turn would 'arm' mast cells with the specific antibody. Activation of mast cells and basophils upon re-exposure to the antigen would then lead to IL-4 production and amplification of the Th2 response (Fig. 2).

*Eosinophils:* While there is a considerable body of evidence to suggest that eosinophils are final effector cells in the pathogenesis of allergic disease and bronchial asthma, mediated largely through the secretion of cationic proteins,[81,82] these cells also have the capacity to synthesize and release a wide array of cytokines. Additionally, eosinophils express various surface markers which suggest that they also may be immunologically competent cells. In this respect, it has recently been demonstrated that eosinophils express CD40L upon activation, suggesting that these cells may be able to provide efficient help to B cells to produce antibodies.[83] Human eosinophils can store IL-5 in the granular matrix which is secreted by either IgA, IgG or IgE dependent mechanisms.[84] Likewise, eosinophils can also produce IL-3 and GM–CSF and as such, eosinophil activation may provide, in an autocrine fashion, its own survival factors. Stimulation of human eosinophils with calcium ionophore also leads to the production of IL-8.[85] However, while IL-8 generation by monocytes or neutrophils can be induced by IL-1β and TNF-α, these cytokines failed to induce IL-8 secretion from eosinophils. Eosinophils can also secrete TGF-α[86] and TGF-β1[87] and as such may account for the eosinophil-derived stimulatory capacity for fibroblast proliferation.[88] This is particularly important in bronchial asthma, where changes in the architecture of the lung may contribute to the irreversibility of this disease. Likewise, human eosinophils synthesize and secrete IL-6[89] which is able to facilitate IL-4 dependent IgE production, and synergizes with IL-3 and GM–CSF for the maturation of multipotential granulocyte progenitors. Interestingly, IL-6 can also promote the secretion of IgA in mucosal tissue, which may subsequently 'arm' eosinophils to secrete their granular contents following activation.

### Th2 inhibitors—novel asthmatic drugs?

Important theoretical questions surround the rational and therapeutic value of Th2 inhibitors as they arise. A central question relates to the role of IL-4 in the longevity and maintenance of Th2 immune responses. While inhibition of IL-4 would prevent the switch B cells to IgE production, it would also inhibit the differentiation of naive T cells to the Th2 phenotype. However, therapeutically this would be an unachievable goal. As allergic individuals retain their sensitivity to allergens over many decades, it might be supposed that IgE-switched B cells and IL-4 committed Th2 cells retain their phenotypes for prolonged periods. If this is the case, continuous exposure to environmental antigens would be expected to reactivate these immune mechanisms in an IL-4 independent manner. If allergic

Table 1. Cytokine production from T cells, metachromatic cells and eosinophils

| Cytokine | CD4 Th1 | CD4 Th2 | CD8 CTTL | CD8 "TH2 like" | Meta-chromatic cells | Eosino-phils |
|---|---|---|---|---|---|---|
| IFN-γ | +++ | – | +++ | + | – | – |
| TNF | +++ | – | + | nd | ++ | ++ |
| IL-2 | +++ | – | +++ | + | – | – |
| IL-3 | ++ | ++ | + | nd | ++ | ++ |
| GM-CSF | ++ | ++ | + | nd | ++ | ++ |
| IL-4 | – | +++ | – | +++ | +++ | ++ |
| IL-5 | – | +++ | – | +++ | ++ | ++ |
| IL-10 | – | +++ | – | +++ | – | – |
| IL-13 | – | +++ | + | nd | ++ | nd |

The panel of cytokines produced by T cells, metachromatic cells and eosinophils (cytokine production: +++, high; ++ intermediate; + detectable; – not detectable; nd not determined).

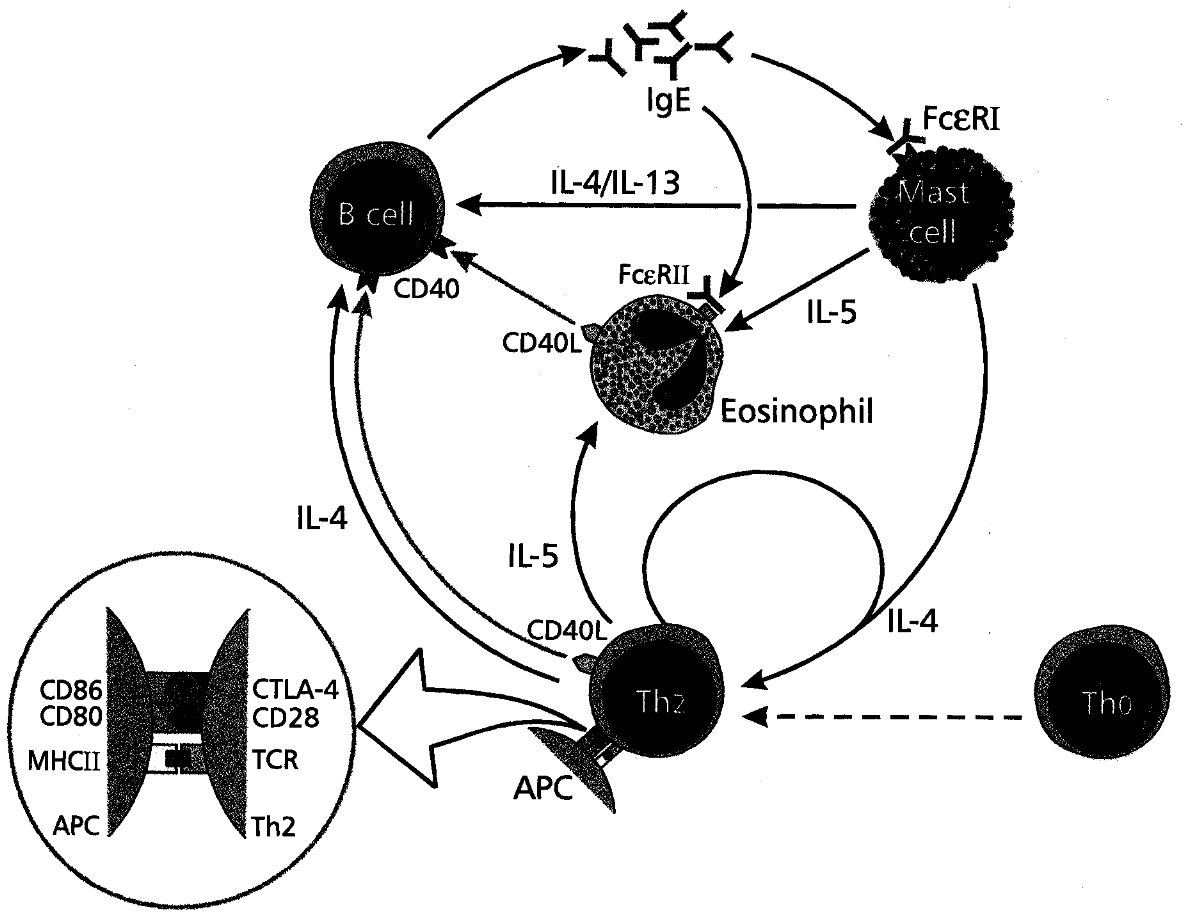

FIG. 1. Hypothetical positive feedback loop for the interaction between mast cells, lymphocytes and eosinophils. Antigen activated Th2 CD4+ T cells, provided with appropriate co-stimulatory signals, secrete IL-4 and express CD40L, and as a consequence instruct B cells to produce IgE. Likewise, Th2 cells produce IL-5 to recruit eosinophils into the lung. Interestingly, activated eosinophils may also express CD40L to help B cell IgE production. IgE can then bind to the FCεRI on mast cells, which, when activated, provide a further source of IL-4 to maintain and amplify the immune response.

immune 'memory' (persisting responsiveness to recall antigens) is IL-4 independent, inhibitors of Th2 cytokine production rather than commitment will prove to be of greater therapeutic value. However, in some murine systems of ongoing IgE synthesis, there is evidence that IL-4 is required for the maintenance of the Th2 phenotype.[90] However, the situation may be more complex in humans, where IL-13 dependent ongoing IgE synthesis may be more important. However, inhibition of IL-4 may offer advantages in steroid resistant asthma by preventing/reversing impaired steroid receptor function[55] and in viral mediated exacerbations of asthma, where IL-4 may be of central importance in switching cytotoxic CD8+ T cells to a Th2 like phenotype.[49,50] Likewise, selective inhibition of IL-5 offers the possibility of selectively suppressing eosinophil infiltration into the airways. However, whether other eosinophil factors such as eotaxin can replace IL-5 in the lung of allergic individuals remains to be determined. Finally, one important point to determine is whether agents that suppress the production of Th2 cytokines from T cells are also effective in suppressing the secretion of cytokines from, for example, mast cells and eosinophils, which may be important sources of cytokines in established chronic asthma.

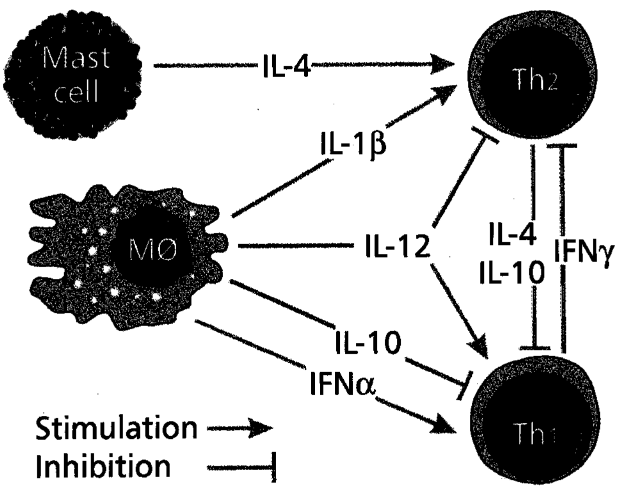

FIG. 2. Interactive cytokine network regulating the commitment of CD4+ cells to a Th1 or Th2 phenotype.

### Conclusions

The cytokines released from Th2 and Th2-like cells are likely to be central to the pathophysiology of asthma and allergy contributing to aberrant IgE production, eosinophilia and, perhaps, mucosal susceptibility to viral infection. IL-4 has emerged as a central target, not only for B cell IgE production, but also in the commitment of both CD4+ and CD8+ T cells to cells with Th2 effector function capable of secreting IL-5 resulting in eosinophilic inflammation. In view of the central role of this cytokine and the evidence that glucocorticoids are unable to modify many IL-4 dependent effects, Th2 inhibitors may prove to be novel therapies for the treatment of bronchial asthma.

### References

1. Djukanovic R, Roche WR, Wilson JW, *et al.* Mucosal inflammation in asthma. *Am Rev Respir Dis* 1990; **142:** 434–457.
2. Corrigan CJ, Hartnell A, Kay AB. T-lymphocyte activation in acute severe asthma. *Lancet* 1988; **1:** 1129–1132.
3. Mosmann TR, Coffman RL. Th1 and Th2 cells: Different patterns of lymphokine secretion lead to different functional properties. *Annu Rev Immunol* 1989; **7:** 145–173.
4. Wierenga EA, Snoek M, Bos JD, Jansen HM, Kapsenberg ML. Comparison of diversity and function of house dust mite-specific lymphocyte clones from atopic and non-atopic donors. *Eur J Immunol* 1990; **20:** 1519–1526.
5. Robinson DS, Hamid Q, Ying S, *et al.* Predominant Th2 like bronchoalveolar T lymphocyte population in atopic asthma. *N Engl J Med* 1992; **326:** 298–304.
6. Bentley AM, Meng Q, Robinson DS, Hamid Q, Kay AB, Durham SR. Increases in activated T lymphocytes, eosinophils and cytokine mRNA expression for interleukin-5 and granulocyte macrophage colony-stimulating factor in bronchial biopsies after allergen inhalation challenge in atopic asthmatics. *Am J Respir Cell Mol Biol* 1993; **8:** 35–42.
7. Hamid Q, Azzawi M, Ying S, *et al.* Expression of mRNA for interleukin-5 in mucosal biopsies from asthma. *J Clin Invest* 1991; **87:** 1541–1546.
8. Linsley PS, Brady W, Grosmaire L, Aruffo A, Damle NK, Ledbetter JA. Binding of the B cell activation antigen B7 to CD28 costimulates T cell proliferation and IL-2 mRNA accumulation. *J Exp Med* 1991; **173:** 721–730.
9. Shahinian A, Pfeffer K, Lee KP, *et al.* Differential T cell costimulatory requirements in CD28-deficient mice. *Science* 1993; **261:** 609–612.
10. Linsley PS, Clark EA, Ledbetter JA. T cell antigen CD28 mediates adhesion with B cells by interacting with activation antigen B7/BB-1. *Proc Natl Acad Sci USA* 1990; **87:** 5031–5035.
11. Linsley PS, Greene JL, Tan P, *et al.* Co-expression and functional cooperation of CTLA-4 and CD28 on activated T lymphocytes. *J Exp Med* 1992; **176:** 1595–1604.
12. Freeman GJ, Borriello F, Hodes RJ, *et al.* Uncovering of functional alternative CTLA-4 counter-receptor in B7-deficient mice. *Science* 1993; **62:** 844–845.
13. Azuma M, Ito D, Yagita H, *et al.* B70 antigen is a second ligand for CTLA-4 and CD28. *Nature* 1993; **366:** 76–79.
14. Lenschow DJ, Sperling AI, Cooke MP, *et al.* Differential up regulation of the B7-1 and B7-2 costimulatory molecules after Ig receptor engagement by antigen. *J Immunol* 1994; **153:** 1990–1997.
15. Boussiotis VA, Freeman GJ, Gribben JG, *et al.* Activated human B lymphocytes express three CTLA-4 counterreceptors that costimulate T-cell activation. *Proc Natl Acad Sci USA* 1993; **90:** 11059–11063.
16. Linsley PS, Brady W, Urnes M, Grosmaire LS, Damle NK, Ledbetter JA. CTLA-4 is a second receptor for the B cell activation antigen B7. *J Exp Med* 1991; **174:** 561–569.
17. Walunas TL, Lenschow DJ, Bakker CY, *et al.* CTLA-4 can function as a negative regulator of T cell activation. *Immunity* 1994; **1:** 405–413.
18. King CL, Stup RJ, Craighead N, June CH, Thyphronitis G. CD28 activation promotes Th2 subset differentiation by human CD4+ cells. *Eur J Immunol* 1995; **25:** 587–595.
19. McArthur JG, Raulet DH. CD28 induced costimulation of T helper type 2 cells mediated by induction of responsiveness to interleukin-4. *J Exp Med* 1993; **178:** 1645–1653.
20. Lu P, di Zhou X, Chen SJ *et al.* CTLA-4 ligands are required to induce an *in vivo* IL-4 response to a gastrointestinal nematode parasite. *J Exp Med* 1994; **180:** 693–698.
21. Lu P, di Zhou X, Chen S-J, *et al.* Requirement of CTLA-4 counter receptors for IL-4, but not IL-10 elevations during a primary systemic *in vivo* immune response. *J Immunol* 1995; **154:** 1078–1087.
22. Ronchese F, Hausmann B, Hubele S, Lane P. Mice transgenic for a soluble form of murine CTLA-4 show enhanced expansion of antigen-specific CD4+ T cells and defective antibody production *in vivo*. *J Exp Med* 1994; **179:** 809–817.
23. Moon HB, Severinson E, Heusser CH, *et al.* Regulation of IgG1 and IgE synthesis by interleukin-4 in mouse B cells. *Scand J Immunol* 1989; **30:** 355–361.
24. Snapper CM, Finkelman F, Paul WE. Regulation of IgG1 and IgE production by interleukin-4. *Immunol Rev* 1988; **102:** 51–75.
25. Snapper CM, Finkelman FD, Paul WE. Differential regulation of IgG1 and IgE synthesis by interleukin-4. *J Exp Med* 1988; **167:** 183–196.
26. Pène J, Rousset F, Brière F, *et al.* IgE production by normal human lymphocytes is induced by interleukin-4 and suppressed by interferons gamma and alpha and prostaglandin E2. *Proc Natl Acad Sci USA* 1988; **85:** 6880–6884.
27. Kopf M, Le Gros G, Bachmann M, Lamers MC, Bluethmann H, Kohler G. Disruption of the murine IL-4 gene blocks Th2 cytokine responses. *Nature* 1993; **362:** 245–247.
28. Von der Weid T, Kopf M, Köhler G, Langhorne J. The immune response to *Plasmodium chabaudi* malaria in interleukin-4-deficient mice. *Eur J Immunol* 1994; **24:** 2285–2293.
29. Zurawski G, de Vries JE. Interleukin 13, an interleukin 4-like cytokine that acts on monocytes and B cells, but not on T cells. *Immunol Today* 1994; **15:** 19–26.
30. Punnonen J, Aversa G, Cocks BG, *et al.* Interleukin 13 induces interleukin 4-independent IgG4 and IgE synthesis and CD23 expression by human B cells. *Proc Natl Acad Sci USA* 1993; **90:** 3730–3734.
31. Brinkman V, Kristofic C. TCR-stimulated naive human CD4+45RO− T cells develop into effector cells that secrete IL-13, IL-5, and IFN-gamma, but not IL-4, help efficient IgE production by B cells. *J Immunol* 1995; **154:** 3078–3087.
32. Basten A, Beeson PB. Mechanisms of eosinophilia. II. Role of the lymphocyte. *J Exp Med* 1970; **131:** 1288–1305.
33. Iwama T, Nagai H, Suda H, Tsuruoka N, Koda A. Effect of murine recombinant interleukin-5 on the cell population in guinea-pig airways. *Br J Pharmacol* 1992; **105:** 19–22.
34. Tominaga A, Takaki S, Koyama N, *et al.* Transgenic mice expressing a B cell growth and differentiation factor gene (interleukin 5) develop eosinophilia and autoantibody production. *J Exp Med* 1991; **173:** 429–437.
35. Coffman RL, Seymour BWP, Hudak S, Jackson J, Rennick D. Antibody to interleukin-5 inhibits helminth-induced eosinophilia in mice. *Science* 1989; **245:** 308–310.
36. Van Oosterhout AJ, Rudof A, Ladenius C, *et al.* Effect of anti-IL-5 and IL-5 on airway hyperreactivity and eosinophils in guinea pigs. *Am Rev Respir Dis* 1993; **147:** 548–552.
37. Yamaguchi Y, Suda T, Ohta S, Tominaga K, Miura Y, Kasahara T. Analysis of the survival of mature human eosinophils: Interleukin-5 prevents apoptosis in mature human eosinophils. *Blood* 1991; **78:** 2542–2547.
38. Her E, Frazer J, Austen KF, Owen WF Jr. Eosinophil hematopoietins antagonize the programmed cell death of eosinophils. Cytokine and glucocorticoid effects on eosinophils maintained by endothelial cell-conditioned medium. *J Clin Invest* 1991; **88:** 1982–1987.
39. Coyle AJ, Le Gros G, Bertrand C, Tsuyuki S, Heusser CH, Kopf M, Anderson GP. Interleukin-4 is required for the induction of lung Th2 mucosal immunity. *Am J Respir Cell Mol Biol* (in press).
40. Tsuyuki S, Bertrand C, Anderson GP, Coyle AJ. Survival of eosinophils in the lung is dependent on IL-5 production from non-CD4+ cells. *Am J Resp Crit Care Med* 1995; **151:** A239.
41. Alam R, Stafford S, Forsythe P, *et al.* RANTES is a chemotactic and activating factor for human eosinophils. *J Immunol* 1993; **150:** 3442–3448.
42. Kameyoshi Y, Dörschner A, Mallet AI, Christophers E, Schröder JM. Cytokine RANTES released by thrombin-stimulated platelet is a potent attractant for human eosinophils. *J Exp Med* 1992; **176:** 587–592.
43. Schall T, Bacon K, Toy K, Goeddel D. Selective attraction of monocytes and T lymphocytes of the memory phenotype by cytokine RANTES. *Nature* 1990; **347:** 669–671.
44. Griffiths-Johnson DA, Collins PD, Rossi AG, Jose PJ, Williams TJ. The chemokine, eotaxin, activates guinea-pig eosinophils *in vitro* and causes their accumulation into the lung *in vivo*. *Biochem Biophys Res Commun* 1993; **197:** 1167–1172.
45. Jose PJ, Griffiths-Johnson DA, Collins PD, *et al.* Eotaxin: A potent eosinophil chemoattractant cytokine detected in a guinea pig model of allergic airways inflammation. *J Exp Med* 1994; **179:** 881–887.
46. Rothenberg ME, Luster AD, Lilly CM, Drazen JM, Leder P. Constitutive and allergen-induced expression of eotaxin mRNA in the guinea pig lung. *J Exp Med* 1995; **181:** 1211–1216.
47. Renz H, Lack G, Saloga J, *et al.* Inhibition of IgE production and normalisation of airway responsiveness by sensitised CD8 T cells in a mouse model of allergen induced sensitisation. *J Immunol* 1994; **152:** 351–360.
48. McMenamin C, Holt PG. The natural immune response to inhaled soluble protein antigens involves major histocompatibility complex (MHC) class I-restricted CD8+ T cell-mediated but MHC class II-restricted CD4+ T cell-dependent immune deviation resulting in selective suppression of immunoglobulin E production. *J Exp Med* 1993; **178:** 889–899.
49. Erard F, Wild MT, Garcia-Sanz JA, Le Gros GG. Switch of CD8 T cells to noncytolytic CD8-CD4- cells that make TH2 cytokines and help B cells. *Science* 1993; **260:** 1802–1805.
50. Coyle AJ, Erard F, Bertrand C, Walti S, Pircher H, Le Gros G. Virus-specific CD8+ cells can switch to interleukin 5 production and induce airway eosinophilia. *J Exp Med* 1995; **181:** 1229–1233.
51. Robinson D, Hamid Q, Ying S, *et al.* Prednisolone treatment in asthma is associated with modulation of bronchoalveolar lavage cell interleukin-4, interleukin-5 and interferon-gamma cytokine gene expression. *Am Rev Respir Dis* 1993; **148:** 401–406.
52. Zubiaga AM, Munoz E, Huber BT. IL-4 and IL-2 selectively rescue Th cell subsets from glucocorticoid-induced apoptosis. *J Immunol* 1992; **149:** 107–112.
53. Kaiser J, Bickel CA, Bochner BS, Schleimer RP. The effects of the potent glucocorticoid budesonide on adhesion of eosinophils to human vascular endothelial cells and on endothelial expression of adhesion molecules. *J Pharmacol Exp Ther* 1993; **267:** 245–249.
54. Le Gros G, Walti S, Bertland C, *et al.* Glucocorticosteroids differentially modulate the production of cytokines from murine Th2 cells. *Am J Resp Crit Care Med* 1995; **151:** A779.
55. Leung DYM, Martin RJ, Szefler SJ, *et al.* Dysregulation of interleukin 4, interleukin 5, and interferon gamma gene expression in steroid-resistant asthma. *J Exp Med* 1995; **181:** 33–40.
56. Kam JC, Szefler SJ, Surs W, Sher ER, Leung DY. The combined effects of IL-2 and IL-4 alter the binding affinity of the glucocorticoid receptor. *J Immunol* 1993; **151:** 3460–3466.
57. Le Gros G, Ben-Sasson SZ, Seder R, Finkelman FD, Paul WE. Generation of interleukin 4 (IL-4)-producing cells *in vivo* and *in vitro*: IL-2 and IL-4 are required for *in vitro* generation of IL-4-producing cells. *J Exp Med* 1990; **172:** 921–929.
58. Swain SL, Weinberg AD, English M, Huston G. IL-4 directs the development of Th2-like helper effectors. *J Immunol* 1990; **145:** 3796–3806.
59. Seder RA, Paul WE, Davis MM, de St. Groth BF. The presence of interleukin 4 during *in vitro* priming determines the lymphokine-producing potential of CD4+ T cells from T cell receptor transgenic mice. *J Exp Med* 1992; **176:** 1091–1098.
60. Bradding P, Feather IH, Howarth PH, *et al.* Interleukin-4 is localized to and released by human mast cells. *J Exp Med* 1992; **176:** 1381–1386.
61. Schmitz J, Thiel A, Kühn R, *et al.* Induction of interleukin 4 (IL-4) expression in T helper (Th) cells is not dependent on IL-4 from non-Th cells. *J Exp Med* 1994; **179:** 1349–1353.
62. Yoshimoto T, Paul WE. CD4pos, NK1.1pos T cells promptly produce interleukin 4 in response to *in vivo* challenge with anti-CD3. *J Exp Med* 1994; **179:** 1285–1295.
63. Fiorentino DF, Bond MW, Mosmann TR. Two types of mouse helper T cells. IV. Th2 clones secrete a factor that inhibits cytokine production by Th1 clones. *J Exp Med* 1989; **170:** 2081–2095.
64. O'Garra A, Stapleton G, Char V, *et al.* Production of cytokines by mouse B cells: B lymphomas and normal B cells produce interleukin 10. *Intern Immunol* 1990; **2:** 821–832.
65. De Waal Malefyt R, Yssel H, Roncarolo M-G, Spits H, De Vries JE. Interleukin-10. *Curr Opinion Immunol* 1992; **4:** 314–320.
66. Fiorentino DF, Zlotnik A, Vieira P, *et al.* IL-10 acts on the antigen presenting cell to inhibit cytokine production by Th1 cells. *J Immunol* 1991; **146:** 3444–3451.
67. Zuany-Amorim C, Haile S, Leduc D, *et al.* Interleukin-10 inhibits antigen-induced cellular recruitment into the airways of sensitized mice. *J Clin Invest* (in press).
68. Gajewski TF, Fitch FW. Anti-proliferative effects of IFN-gamma in immune regulation. IFN-gamma inhibits the proliferation of Th2 but not Th1 murine helper T lymphocytes clones. *J Immunol* 1988; **140:** 4245–4253.
69. Nakajima H, Iwamoto I, Yoshisa S. Aerosolised recombinant interferon-gamma prevents antigen induced eosinophil recruitment in mouse trachea. *Am Rev Respir Dis* 1993; **148:** 1102–1104.
70. Seder R, Gazzinelli AR, Sher A, Paul WE. Interleukin 12 acts directly on CD4+ T cells to enhance priming for interferon gamma production and diminishes interleukin 4 inhibition of such priming. *Pro Natl Acad Sci USA* 1993; **90:** 10188–10192.
71. Sypek JP, Chung CL, Major SE, *et al.* Resolution of a cutaneous leishmaniasis: Interleukin-12 initiates a protective T helper Type 1 immune response. *J Exp Med* 1993; **177:** 1797–1803.
72. Finkelman FD, Madden KB, Cheever AW, *et al.* Effects of IL-12 on immune responses and host protection in mice infected with intestinal nematode parasites. *J Exp Med* 1994; **179:** 1563–1572.
73. Shu U, Demeure CE, Byun D, Podlaski F, Stern AS, Delespesse G. Interleukin 12 exerts a differential effect on the maturation of neonatal and adult human CD45RO-CD4T cells. *J Clin Invest* 1994; **94:** 1352–1358.
74. Finkelman FD, Svetic A, Gresser I, *et al.* Regulation by interferon-$\alpha$ of immunoglobulin isotype selection and lymphokine production in mice. *J Exp Med* 1991; **176:** 1179–1188.
75. Pene J, Rousset F, Briere F, *et al.* IgE production by normal human lymphocytes is induced by interleukin 4 and suppressed by interferons gamma and alpha and prostaglandin E2. *Proc Natl Acad Sci USA* 1988; **85:** 6880–6884.
76. Brinkmann V, Heusser C, Baer C, Kilchherr E, Erard F. Interferon alpha suppresses the capacity of T cells to help antibody production by human B cells. *J Interferon Res* 1992; **12:** 267–274.
77. Finkelman FD, Holmes J, Katona IM, *et al.* Lymphokine control of *in vivo* immunoglobulin isotype selection. *Annu Rev Immunol* 1990; **8:** 303–333.
78. Brinkmann V, Geiger T, Alkan S, Heusser CH. Interferon-$\alpha$ increases the frequency of interferon gamma-producing human CD4+ T cells. *J Exp Med* 1993; **178:** 1655–1663.
79. Aoki I, Kinzer C, Shirai A, Paul WE, Klinman DM. IgE receptor-positive non-B/non-T cells dominate the production of interleukin 4 and interleukin 6 in immunized mice. *Proc Natl Acad Sci USA* 1995; **92:** 2534–2538.
80. Burd PR, Thompson WC, Max EE, Mills FC. Activated mast cells produce interleukin 13. *J Exp Med* 1995; **181:** 1373–1380.
81. Coyle AJ, Ackerman SJ, Irvin CG. Cationic proteins induce airway hyperresponsiveness dependent on charge interactions. *Am Rev Respir Dis* 1993; **147:** 896–900.
82. Coyle AJ, Perretti F, Manzini S, Irvin CG. Cationic protein-induced sensory nerve activation: Role of substance P in airway hyperresponsiveness and plasma protein extravasation. *J Clin Invest* 1994; **94:** 2301–2306.
83. Gauchat J-F, Henchoz S, Fattah D, *et al.* CD40 ligand is functionally expressed on human eosinophils. *Eur J Immunol* 1995; **25:** 863–865.
84. Dubucquoi S, Desreumaux P, Janin A, *et al.* Interleukin 5 synthesis by eosinophils: Association with granules and immunoglobulin-dependent secretion. *J Exp Med* 1994; **179:** 703–708.
85. Braun RK, Franchini M, Erard F, *et al.* Human peripheral blood eosinophils produce and release interleukin-8 on stimulation with calcium ionophore. *Eur J Immunol* 1993; **23:** 956–960.
86. Wong DTW, Weller P, Galli SJ, *et al.* Human eosinophils express transforming growth factor alpha. *J Exp Med* 1990; **172:** 673–681.
87. Wong DTW, Elovic A, Matossian K, *et al.* Eosinophils from patients with blood eosinophilia express transforming growth factor $\beta$1. *Blood* 1991; **78:** 2707–2707.
88. Pincus SH, Ramesh KS, Wyler DJ. Eosinophils stimulate fibroblast DNA synthesis. *Blood* 1987; **70:** 572–574.
89. Hamid Q, Barkans J, Meng Q, *et al.* Human eosinophils synthesize and secrete interleukin-6, *in vitro*, *Blood* 1992; **180:** 1496–1501.
90. Finkelman FD, Katona IM, Urban Jr JF, *et al.* IL-4 is required to generate and sustain *in vivo* IgE responses. *J Immunol* 1988; **141:** 2335–2341.

ACKNOWLEDGMENTS. The authors would like to thank their colleagues, Drs G. P. Anderson, V. Brinkman, Ch. Heusser, F. Erard and M. Bray, in the Dept. of Asthma and Allergy research at CIBA, Basel, for their helpful discussions and insights on many of the ideas expressed in this review; and to Mrs J. Tsuyuki for excellent secretarial help.

**Received 19 May 1995; accepted 22 May 1995**
